# The Influence of New Hydrophobic Silica Nanoparticles on the Surface Properties of the Films Obtained from Bilayer Hybrids

**DOI:** 10.3390/nano7020047

**Published:** 2017-02-20

**Authors:** Cristian Petcu, Violeta Purcar, Cătălin-Ilie Spătaru, Elvira Alexandrescu, Raluca Şomoghi, Bogdan Trică, Sabina Georgiana Niţu, Denis Mihaela Panaitescu, Dan Donescu, Maria-Luiza Jecu

**Affiliations:** R & D National Institute for Chemistry and Petrochemistry—ICECHIM, Polymers Department, Spl. Independentei 202, 6th District, 060021 Bucharest, Romania; cpetcu@icf.ro (C.P.); spataru.catalin@gmail.com (C.-I.S.); elviraalexandrescu@yahoo.com (E.A.); r.somoghi@gmail.com (R.S.); trica.bogdan@gmail.com (B.T.); sabina.nitu@yahoo.com (S.G.N.); panaitescu@icf.ro (D.M.P.); ddonescu@chimfiz.icf.ro (D.D.); jecu.luiza@icechim.ro (M.-L.J.)

**Keywords:** sol–gel process, silica nanoparticles, nanostructuration, organic–inorganic hybrids, trialkylmonoalkoxysilanes

## Abstract

Ultra-hydrophobic bilayer coatings on a glass surface were fabricated by sol–gel process using hexadecyltrimethoxysilane (C_16_TMS) and tetramethoxysilane (TMOS) (1:4 molar ratio) as precursors. After coating, silica nanoparticles (SiO_2_ NPs) functionalized with different mono-alkoxy derivatives (methoxytrimethylsilane, TMeMS; ethoxydimethylvinylsilane, DMeVES; ethoxydimethylphenylsilane, DMePhES; and methoxydimethyloctylsilane, DMeC_8_MS) were added, assuring the microscale roughness on the glass surface. Influences of the functionalized SiO_2_ NPs and surface morphology on the hydrophobicity of the hybrid films were discussed. The successful functionalization of SiO_2_ NPs with hydrophobic alkyl groups were confirmed by Fourier transform infrared spectroscopy (FTIR). The thermal stability of hydrophobic SiO_2_ NPs showed that the degradation of the alkyl groups takes place in the 200–400 °C range. Bilayer coating with C_16_TMS/TMOS and SiO_2_ NPs modified with alkoxysilane substituted with C_8_ alkyl chain (SiO_2_ NP-C_8_) has micro/nano structure. Hydrophobicity of functionalized SiO_2_ NPs-C_8_ and its higher degree of nanometer-scale roughness gave rise to ultra-hydrophobicity performance for bilayer coating C_16_TMS/TMOS + SiO_2_ NPs-C_8_ (145°), compared to other similar hybrid structures. Our synthesis method for the functionalization of SiO_2_ NPs is useful for the modification of surface polarity and roughness.

## 1. Introduction

To obtain highly hydrophobic surfaces, a large number of review-type studies have been designed. The main properties of highly hydrophobic surfaces are weak water adhesion and self-cleaning behavior in the presence of wetting phenomenon. In most cases, these surfaces are used in construction, dyes, windshield and window manufacturing, headlights, automobiles, solar panels, good thermal transfer pipe-lines, sensors, etc. [1,2,3,4].

A large and diverse number of methods to obtain highly hydrophobic surfaces have been reported, such as multi-step chemical methods and physical methods that create roughness [1,5,6]. Various functional silane precursors—polymethylhydrosiloxane, poly(vinyl chloride), fluorinated methacrylate, and mercapto functional monomers were used in most situations of sol–gel method in order to fabricate superhydrophobic surfaces [7].

From analysis of the published data, two approaches for obtaining hydrophobic surfaces from particles and binder were notable: mixing the two phases [8,9,10,11], or deposition of particles over films obtained from different film-forming procedures [12,13,14,15,16,17,18,19,20,21,22]. In this way, the possibility of obtaining superhydrophobic surfaces (contact angle > 160°) by mixing silica nanoparticles (SiO_2_ NPs) with a binder obtained from tetraethoxysilane (TEOS) and a fluorosilane (FAS) through acid-catalyzed sol–gel process were reported [8,9]. In the case of depositing SiO_2_ NPs over different supports, ultrahydrophobic surfaces were obtained by functionalizing the fillers with hydrocarbon chains [12,13,14,15].

Thereby, functionalizing silica particles with trichlorosilanes led to the formation of surfaces whose hydrophobicity increase with increasing alkyl chain length. When n-octadecyltrichlorosilane (ODTS) was used for hydrophobic process, a contact angle (CA) of 162° was achieved [12]. Xiu et al. [13] report the effect of surface hydrophobicity on the measured contact angles on the rough surfaces. It was demonstrated that superhydrophobic surfaces can be obtained using silica nanoparticles functionalized with silanes containing different hydrocarbon or fluorocarbon chains.

Yilgor et al. [14] synthesized SiO_2_ NP depositions with controllable hydrophobicity using mixtures of hydrophilic silica with hydrophobic silica (modified with trimethylchlorosilanes) onto the polymer surface. Another research group obtained superhydrophobic films using SiO_2_ nanoparticles (synthesized with TEOS in emulsions containing toluene and a mixture of neutral and anionic surfactants) deposited on glass substrates [15]. Superhydrophobic silica layers obtained by thermally treating a mixture that contained dual-sized polystyrene particles were reported [16]. The final process of hydrophobization was accomplished in dodecafluoroheptyl-methyldimethoxysilane vapors, showing excellent superhydrophobic property of silica film. Additionally, a covalent bond between the two phases can represent a special way of ensuring the deposition of silica particles over a polymer matrix [17,18,19,20]. “Layer-by-layer” (LBL) gradual deposition of dual-sized silica particles on glass functionalized with NH_3_^+^ groups and modified with dodecyltrichlorosilane was shown in [21]. Similar CA values were obtained for LBL depositions [22] of SiO_2_ NP dispersions over polymer layers with polyalkylamine hydrochloride.

This paper will focus on the aspect of synthesis, size-dependent properties, and modification of silica nanoparticles (SiO_2_ NPs) by sol–gel method using different alkoxysilanes (substituted with dimethyl and methyl, vinyl, phenyl, or octyl alkyl group) toward the preparation of bilayer coatings on a glass surface. The influence of the functionalized SiO_2_ NPs with mono-alkoxy derivatives (methoxytrimethylsilane, TMeMS; ethoxydimethylvinylsilane, DMeVES; ethoxydimethylphenylsilane, DMePhES; methoxydimethyloctylsilane, DMeC_8_MS) on the hydrophobicity is studied. The comparative analysis brings new information regarding the interactions between the alkyl group from the functionalized SiO_2_ NPs layer and hexadecyltrimethoxysilane (C_16_TMS)/tetramethoxysilane (TMOS) hybrid film. Our synthesis method is useful for the modification of surface polarity and wettability. The resultant coatings are characterized through various techniques, including dynamic light scattering (DLS), Fourier transform infrared spectroscopy (FTIR), thermogravimetric analysis (TGA), environmental scanning electron microscopy (ESEM), transmission electron microscopy (TEM), atomic force microscopy (AFM), and water contact angles (CA).

## 2. Results and Discussion

For increasing hydrophobicity, it is well known that the only method which does not involve any additional oligomerization reactions is the use of monochloro- or monoalkoxysilanes [4,5,23]. In the case of functionalizing silica particles or dispersed layered silicates, silanes with two or three reactive groups can induce aggregation of the fillers due to the formation of multiple covalent bonds [24,25]. The only difficulty when the functionalization is performed with monofunctional silanes is weak reactivity [23,26]. In order to increase the reactivity, in this study, ultrasonication was used throughout the entire reaction and heating process [27].

**Figure 1:**
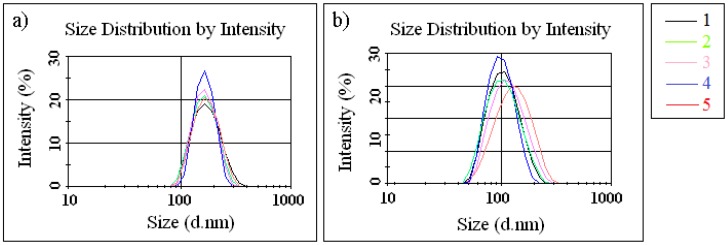
The average particle size of pristine silica nanoparticles (SiO_2_ NPs) and functional SiO_2_ NPs dispersed in: (**a**) ethanol and (**b**) methylene chloride.

The functionalization reaction was confirmed by measuring the average diameters of the silica particles dispersed in ethanol (Figure 1a) and methylene chloride (Figure 1b). The highest value was obtained for the silica functionalized with long alkyl chain (*sample 5*, Figure 1b). These results agree with previous results regarding mesoporous organized silica functionalization. When the auxiliary organic species are added to the reaction gel, they are solubilized inside the hydrophobic regions of material, causing an increase in the pore size of the final product [28]. Experiments have shown that the SiO_2_ NPs functionalized with short alkyl groups in the reaction mixture lead to a smaller particle size compared to pristine SiO_2_ NPs only as a source of silica. These findings indicate that the addition of organosilane with short alkyl group affect the nucleation process and lead to a larger number of formed nuclei. Thus, smaller particle size is obtained (*samples 2*–*4*). The average particle size increased for *sample 5* (see Figure 1b), and this effect can be caused by steric hindrance and is seen notably in branched chain (longer alkyl chain leads to lower hydrolysis rate). The stability of the particle dispersion can be achieved by steric stabilization, and is almost attained by proper particle surface functionalization.

**Figure 2:**
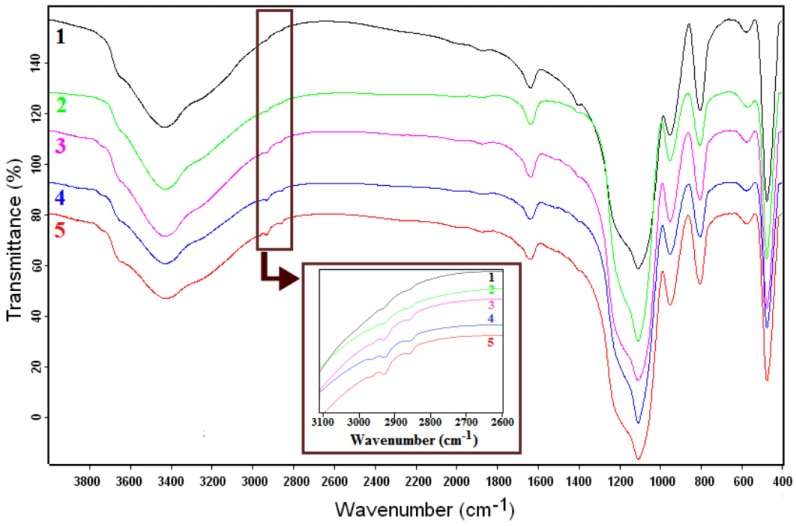
Fourier transform infrared (FTIR) spectra of dried SiO_2_ NPs.

The existence of alkyl groups on the silica surface is confirmed by FTIR spectra (Figure 2), in agreement with previously published data [12,27]. For all samples, the Si-O-Si symmetric and asymmetric bands located at ~800 cm^−1^ and ~1100 cm^−1^, respectively, and the band at 950 cm^−1^ corresponding to the Si-O group can be observed [29,30,31]. The intensity of the C-H stretching bands (peaks observed at 2923 cm^−1^ and 2863 cm^−1^, respectively) is highest for the silica modified with longest alkyl chains (C_8_) (*sample 5*). Substitution with a shorter alkyl chain is characterized by a change in the absorption maximum and a reduced intensity of the bands. This phenomenon can occur due to a different conformation of the alkyl chains [30] and a lower concentration [12] (*samples 2*–*4*). In the case of methyl group substitution (*sample 1*), no significant absorption can be observed in the mentioned wavelength domain.

Chemical modifications with monoalkoxysilanes after the reaction were outlined by the thermal degradation of the new reaction products. In Table 1, the TGA data are given for the three temperature steps. From previously published studies [12,27], the weight loss in the 25–200 °C interval corresponds to the vaporization of the solvent adsorbed on the particles [32,33]. The thermo-oxidative degradation of the alkyl groups caused by a hydrophobic process takes place in the 200–400 °C range. The last degradation step—from 400 to 700 °C—corresponds to the condensation reaction of the OH groups from the partially modified silica surfaces [34].

**Figure 3:**
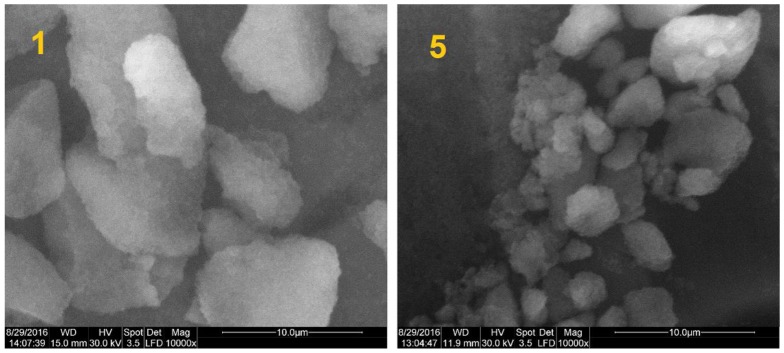
Environmental scanning electron microscopy (ESEM) images of dried pristine SiO_2_ NPs (*sample 1*) and dried SiO_2_ NPs modified with long alkyl chain (*sample 5*), dispersed in ethanol.

**Figure 4:**
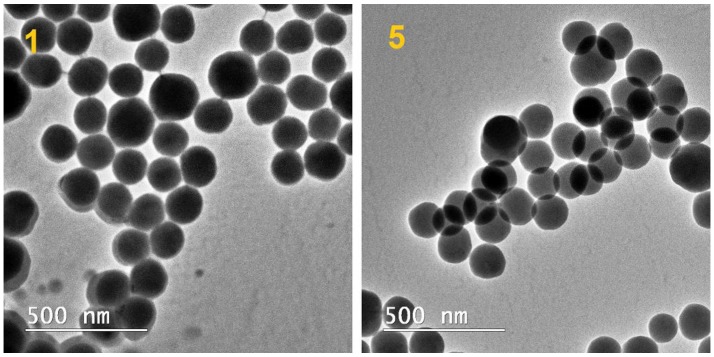
TEM images of dried pristine SiO_2_ NPs (*sample 1*) and dried SiO_2_ NPs modified with long alkyl chain (*sample 5*), dispersed in ethanol.

The morphology and size of the obtained particles have been examined by environmental scanning electron microscopy and transmission electron microscopy. ESEM and TEM analyses (Figure 3 and Figure 4) were performed to confirm the DLS measurements. For this purpose, dried pristine SiO_2_ NP (*sample 1*) and dried SiO_2_ NP modified with C_8_ (*sample 5*) dispersed in ethanol were analyzed. For *sample 1*, it can be observed that not all particles are spherical, and some of them are irregular. SiO_2_ NPs with a size range of 140–170 nm were obtained. Synthesized silica nanoparticles functionalized with long alkyl chain are spherical in shape with diameter of ~150 nm (*sample 5*). 

**Figure 5:**
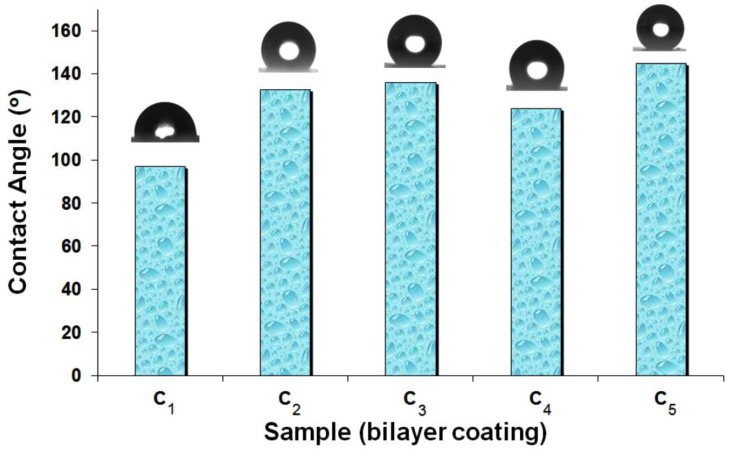
Contact angle of the bilayer coatings (see Table 3).

**Figure 6:**
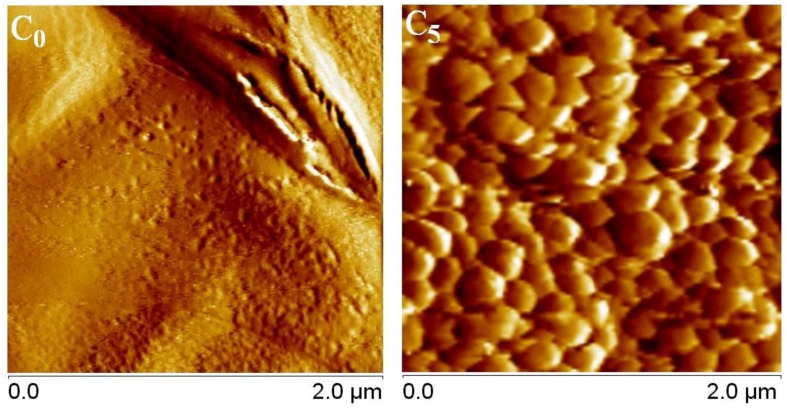
Atomic force microscopy (AFM) topographic images of structured coatings: monolayer hybrid hexadecyltrimethoxysilane/ tetramethoxysilane (C_16_TMS/TMOS) film (*coating C*_0_), and bilayer coating (C_16_TMS/TMOS + 0.01 g of SiO_2_ NPs functionalized with methoxydimethyloctylsilane (DMeC_8_MS) dispersed in 1 mL EtOH, *coating C*_5_).

The wetting ability and surface roughness changes of the bilayer coatings were evaluated by contact angle (CA) measurement and AFM topography, respectively, and the results are as shown in Figure 5 and Figure 6. Figure 5 shows the contact angle (CA) of a 5 μL water droplet on the bilayer coatings. Comparing the CA values, a maximum value (145°) was obtained for bilayer coating with C_16_TMS/TMOS and 0.01 g of SiO_2_ NPs functionalized with DMeC_8_MS dispersed in 1 mL EtOH (*coating C*_5_). This observation means that the coated surface is rough on the micro/nanometer scale. For *coatings C*_1_–*C*_4_, the degree of roughness was not sufficient, and ultra-hydrophobicity was not achieved. This effect can be explained by considering the chemical reactivity of functionalized silica nanoparticles that were not strongly fixed to the first layer (C_16_TMS/TMOS). The surface hydrophobicity is in fact due to the presence of functional hydrophobic groups that start to adhere to the base silicate matrix.

Figure 6 shows AFM topographic images of C_16_TMS/TMOS monolayer hybrid film (*coating C*_0_), and of bilayer coating (C_16_TMS/TMOS + 0.01 g of SiO_2_ NPs functionalized with DMeC_8_MS dispersed in 1 mL EtOH, *coating C*_5_). As can be observed, the starting C_16_TMS/TMOS hybrid film surface is quite smooth. *Sample 5* presents a rough surface, which is attributed to the adhesion between functionalized SiO_2_ NPs with long alkyl group and Si-O- groups from the C_16_TMS/TMOS substrate. The higher degree of nanometer-scale roughness gave rise to ultra-hydrophobicity.

## 3. Experimental Section

### 3.1. Materials

Tetraethylorthosilicate (TEOS, 98%, Aldrich, Steinheim, Germany), methoxytrimethylsilane (TMeMS, 97%, Fluka, Steinheim, Germany), ethoxydimethylvinylsilane (DMeVES, 97%, Alfa Aesar, Swedesboro, NJ, USA), ethoxydimethylphenylsilane (DMePhES, 97%, Fluka, Steinheim, Germany), methoxydimethyloctylsilane (DMeC_8_MS, 98%, Aldrich, Steinheim, Germany), methoxydimethyloctadecylsilane (DMeC_18_MS, 90%, Aldrich, Steinheim, Germany), hexadecyltrimethoxysilane (C_16_TMS, 85%, Aldrich, Steinheim, Germany), ethanol (reagent grade, CHIMREACTIV SRL), ammonia (reagent grade, 32 wt. %, Scharlau, Sentmenat, Spain), hydrochloric acid (reagent grade, 37 wt. %, Riedel, Seelze, Germany), toluene (reagent grade, CHIMREACTIV SRL).

### 3.2. Preparation of Silica Nanoparticles (SiO_2_ NPs) 

The synthesis of hydrophobic modified SiO_2_ nanoparticles (SiO_2_ NPs) was realized in two steps by a method adapted from a study published by Kulkarni et al. [11].

#### 3.2.1. Synthesis of the Pristine Silica Nanoparticles

TEOS (32.5 g) and 125 mL absolute ethanol (EtOH) were initially introduced in a three-neck round-bottom flask with a mechanical stirrer (400 rot/min) and a reflux condenser. Under stirring, a mixture of 75 mL NH_4_OH (25%) and 570 mL absolute EtOH was added for 2 h. After addition of ~100 mL of ammonia solution, the mixture turned opalescent. At the end of the addition of the ammonia solution, the mixture was kept under stirring for another two hours. The mixture was put in Petri dishes, and the volatile compounds were allowed to evaporate at ambient temperature and in vacuum at 60 °C. After drying, 9.33 g SiO_2_ NPs were obtained (*sample 1*) and used for their functionalization with various mono-alkoxy derivatives.

#### 3.2.2. Functionalization of the Silica Nanoparticles with Various Mono-Alkoxy Derivatives 

Different alkoxysilanes: methoxytrimethylsilane (TMeMS), ethoxydimethylvinyl silane (DMeVES), ethoxydimethylphenylsilane (DMePhES), and methoxydimethyloctylsilane (DMeC_8_MS) were used to functionalize the SiO_2_ NPs by sol–gel process. SiO_2_ (1.5 g, synthesized in Section 3.2.1) and 100 mL of toluene (dried on molecular sieves and deoxygenated with nitrogen) were added in a three-neck round-bottom flask provided with mechanical stirrer and reflux condenser. The entire mixture was heated at 50 °C under continuous stirring. Then, 7.5 mmoles of mono-alkoxy derivatives substituted with dimethyl and methyl, vinyl, phenyl, octyl, and octadecyl were added to the heated mixture. The mixture was kept at 50 °C under ultrasonication (in an ultrasonic bath) for 4 h. Subsequently, the mixture was dried in air at room temperature (*samples 2*–*5*) (see Table 2). The resulting functionalized silica particles and corresponding coatings are described in Table 3.

### 3.3. Fabrication of the Bilayer Coatings on the Glass Substrate

#### 3.3.1. Preparation of the First Coating Layer

The glass substrates were firstly covered with acidic solution (prepared in a similar way to that previously reported [6]) containing hexadecyltrimethoxysilane (C_16_TMS) and tetramethoxysilane (TMOS). Then, 1.21 g of C_16_TMS and the amount of TMOS required to obtain a molar ratio of 1/4 were dissolved under continuous stirring in 8.04 mL EtOH and then heated at 40 °C. When the final temperature was achieved, 1.04 mL of HCl (0.1 N solution) was added. The solution was stirred for another 90 min. The molar ratios used for the reaction mixture were as follows: C_16_TMS:TMOS:EtOH:H_2_O:HCl = 1:4:50:19:0.03. The obtained hybrid solution was deposited onto a glass slide by draw down sample coating with the manual applicator. The resultant first layer was left to dry at room temperature for 24 h (*coating C*_0_).

#### 3.3.2. Preparation of the Second Coating Layer

A second solution (0.01 g of dried functionalized SiO_2_ nanoparticles dispersed in 10 mL EtOH and mixed for approximately 2 h) was deposited over the *first layer* in order to obtain *bilayer coatings* (*coatings C*_1_–*C*_5_).

The resultant functionalized silica particles and corresponding coatings are described in Table 3.

### 3.4. Characterization Methods

The initial solutions were examined by dynamic light scattering (DLS) to measure the particle diameters (Zetasizer, Malvern Nano ZS). The solutions were diluted in ethanol and methylene chloride (0.1 mL sample dissolved in 25 mL solvent and ultrasonicated 5 min.).

FTIR spectra of dried samples were obtained using a Fourier transform infrared spectrophotometer (Tensor 37 from Bruker). The samples were ground with KBr and pressed to form a disc for FTIR scanning. For all scans, the spectra were collected over the wavenumber range of 400–4000 cm^−1^, with a resolution of 4 cm^−1^. 

TGA analyses of dried functionalized SiO_2_ NPs were performed in N_2_ (10 °C/min), 25–700 °C range, using a TA TGA Q5000 IR instrument.

The morphology of silica particles was imaged using environmental scanning electron microscopy (ESEM) (FEI QUANTA 200) and by Transmission Electron Microscopy (TEM), employing a Tecnai^TM^ G2 F20 TWIN Cryo-TEM instrument (FEI Company) at 200 kV acceleration voltages. The ESEM images were obtained in the low-vacuum mode. The samples were deposited on a conductive stub and dried at room temperature. For TEM analysis, the samples were observed directly without further staining to improve contrast. A droplet of diluted sample was poured on a carbon film-coated copper grid and allowed to dry in air at room temperature.

Contact angles of bilayer coatings were determined using a CAM 200 Contact Angle Tensiometer instrument from KSV Instruments equipped with a digital camera that was connected to a PC. Water was used as the liquid for the contact angle measurements. The contact angles were calculated from the drop images after the shape accentuation, radius, and string reading were determined.

The roughness of bilayer coatings were studied using an atomic force microscope (AFM) from Bruker, Santa Barbara, equipped with a Nanoscope V controller and a MultiMode head. The equipment was operated in tapping mode using an etched silicon tip (nominal radius 8 nm), a cantilever length of 225 μm, and a resonant frequency of approximately 75 kHz. AFM measurements were performed at room temperature with a scan rate of 1 Hz and a scan angle of 0°.

## 4. Conclusions

Bilayer coatings on glass surfaces were fabricated using functionalized silica nanoparticles obtained by sol–gel process (ultrasonication at 50 °C). FTIR spectra confirmed the existence of the alkyl groups over the silica surface, in agreement with previously published data. The intensity of the C-H stretching bands was the highest in the case of silica modified with long alkyl chain (*C*_8_). Substitution with a shorter alkyl chain was characterized by a modified absorption maximum and a reduced intensity of the bands; methyl group substitution proved to have no significant absorption that could be observed in the mentioned wavelength domain. The thermal stability of the dried silica nanoparticles showed that the weight loss occurred stepwise, and indicated that silica nanoparticles began to lose hydrophobicity when heated in the 200–400 °C range. ESEM and TEM analyses of samples dried and dispersed in ethanol showed that the silica nanoparticles are mostly spherical in shape, with diameter of ~150 nm. The AFM measurements showed that the starting C_16_TMS/TMOS hybrid film surface has smooth surface. Bilayer coating with SiO_2_ NPs modified with C_8_ chain present a higher degree of nanometer-scale roughness (CA = 145°). The obtained bilayer coatings can be useful and helpful to construct artificial anti-wetting surfaces for numerous practical applications.

## Figures and Tables

**Table 1:** Thermal degradation of dried SiO_2_ NPs.

Sample No.	ΔG (% grav.)/T_max_ (°C)			
	25–200 °C	200–400 °C	400–700 °C	Residue (%)
1	7.0/47.3	2.2/277.4	2.5/461.0	88.3
2	6.5/48.6	2.8/263.0	2.4/451.0	88.3
3	6.4/48.2	2.8/272.0	2.5/462.1	88.3
4	6.3/51.4	2.8/293.0	2.7/453.0	88.2
5	6.3/50.4	3.1/257.8	2.5/435.0	88.2

**Table 2:** Synthesis conditions for functionalization of silica.

Sample No.	Synthesis	
	Pristine SiO_2_ Particles (g)	R(CH_3_)_2_SiOR′ *
1	1.5	0
2	1.5	Me_3_SiOMe
3	1.5	VMe_2_SiOEt
4	1.5	PhMe_2_SiOEt
5	1.5	C_8_Me_2_SiOMe

* R = –CH_3_ (Me), –CH_2_=CH_2_– (V), C_6_H_5_– (Ph), CH_3_–(CH_2_)_7_– (C_8_); R′ = –CH_3_ (Me), –CH_2_–CH_3_ (Et).

**Table 3:** Composition of functionalized silica particles and the corresponding coatings.

Sample No.	Composition	Corresponding Coatings
-	-	C_0_
1	Pristine SiO_2_ NP	C_1_
2	SiO_2_ NP functionalized with TMeMS	C_2_
3	SiO_2_ NP functionalized with DMeVES	C_3_
4	SiO_2_ NP functionalized with DMePhES	C_4_
5	SiO_2_ NP functionalized with DMeC_8_MS	C_5_

## Supplementary Materials

The following are available online at http://www.mdpi.com/2079-4991/7/2/47/s1, Table S1: DLS results for functionalized silica nanoparticles.
