# Supplementary material for: The Influence of New Hydrophobic Silica Nanoparticles on the Surface Properties of the Films Obtained from Bilayer Hybrids
Source: Nanomaterials (Basel). 2017 Feb 20;7(2):47. doi: 10.3390/nano7020047 (PMC5333032; doi:10.3390/nano7020047)
Supplement: Supplementary file 1 [file nanomaterials-07-00047-s001.pdf]

Supplementary Material

Table S1. DLS results for functionalized silica nanoparticles

Sample no.	Synthesis		D (nm)	D (nm)
	pristine SiO2 particles (g)	$R(CH_3)_2SiOR'$ *	(using EtOH)	(using CH_2Cl_2)
1	1.5	0	167	100
2	1.5	Me_3SiOMe	157	97
3	1.5	VMe_2SiOEt	158	110
4	1.5	$PhMe_2SiOEt$	163	95
5	1.5	C_8Me_2SiOMe	163	131

* R = $-CH_3$ (Me), $-CH_2=CH_2-$ (V), C_6H_{5-} (Ph), $CH_3-(CH_2)_7-$ (C_8); R' = $-CH_3$ (Me), $-CH_2-CH_3$ (Et).

© 2017 by the authors; licensee MDPI, Basel, Switzerland. This article is an open access article distributed under the terms and conditions of the Creative Commons Attribution (CC BY) license (http://creativecommons.org/licenses/by/4.0/).
